# Plasma Vitamin D Deficiency Is Associated with Poor Sleep Quality and Night-Time Eating at Mid-Pregnancy in Singapore

**DOI:** 10.3390/nu9040340

**Published:** 2017-03-29

**Authors:** Tuck Seng Cheng, See Ling Loy, Yin Bun Cheung, Shirong Cai, Marjorelee T. Colega, Keith M. Godfrey, Yap-Seng Chong, Kok Hian Tan, Lynette Pei-Chi Shek, Yung Seng Lee, Ngee Lek, Jerry Kok Yen Chan, Mary Foong-Fong Chong, Fabian Yap

**Affiliations:** 1 Department of Paediatrics, KK Women’s and Children’s Hospital, 100, Bukit Timah Road, Singapore 229899, Singapore; tscheng1988@gmail.com (T.S.C.); lek.ngee@singhealth.com.sg (N.L.); 2 School of Public Health, Li Ka Shing Faculty of Medicine, The University of Hong Kong, 7 Sassoon Road, Pokfulam, Hong Kong, China; 3 Department of Reproductive Medicine, KK Women’s and Children’s Hospital, 100, Bukit Timah Road, Singapore 229899, Singapore; loy.see.ling@kkh.com.sg (S.L.L.); jerrychan@duke-nus.edu.sg (J.K.Y.C.); 4 Duke-NUS Medical School, 8 College Road, Singapore 169857, Singapore; 5 Center for Quantitative Medicine, Duke-NUS Medical School, 8 College Road, Singapore 169857, Singapore; yinbun.cheung@duke-nus.edu.sg; 6 Tampere Center for Child Health Research, University of Tampere and Tampere University Hospital, Kalevantie 4, Tampere 33100, Finland; 7 Department of Obstetrics & Gynaecology, Yong Loo Lin School of Medicine, National University of Singapore, 1E Kent Ridge Rd 119228, NUHS Tower Block, Level 11, Singapore 117597, Singapore; obgcais@nus.edu.sg (S.C.); obgcys@nus.edu.sg (Y.-S.C.); 8 Singapore Institute for Clinical Sciences, Agency for Science, Technology and Research, (A*STAR), 30 Medical Drive, Singapore 117609, Singapore; marjorelee_colega@sics.a-star.edu.sg (M.T.C.); yung_seng_lee@nuhs.edu.sg (Y.S.L.); mary_chong@sics.a-star.edu.sg (M.F.-F.C.); 9 MRC Lifecourse Epidemiology Unit, University of Southampton, University Road, Southampton SO17 1BJ, UK; kmg@mrc.soton.ac.uk; 10 NIHR Southampton Biomedical Research Centre, University of Southampton and University Hospital Southampton NHS Foundation Trust, University Road, Southampton SO17 1BJ, UK; 11 Maternal Fetal Medicine, KK Women’s and Children’s Hospital, 100, Bukit Timah Road, Singapore 229899, Singapore; tan.kok.hian@kkh.com.sg; 12 Department of Paediatrics, Yong Loo Lin School of Medicine, National University of Singapore, 1E Kent Ridge Rd 119228, NUHS Tower Block, Level 11, Singapore 117597, Singapore; lynette_shek@nuhs.edu.sg; 13 Khoo Teck Puat-National University Children’s Medical Institute, National University Hospital, National University Health System, 5 Lower Kent Ridge Road, Singapore 119074, Singapore; 14 Saw Swee Hock School of Public Health, National University of Singapore, 1E Kent Ridge Rd 119228, NUHS Tower Block, Level 11, Singapore 117597, Singapore; 15 Clinical Nutrition Research Centre, Singapore Institute for Clinical Sciences (SICS), Agency for Science, Technology and Research (A*STAR), 14 Medical Drive #07-02, MD 6 Building, Singapore 117599, Singapore; 16 Lee Kong Chian School of Medicine, Nanyang Technological University, 50 Nanyang Ave, Singapore 639798, Singapore

**Keywords:** vitamin D, behavioral circadian rhythms, sleep quality, predominantly night-time eating

## Abstract

Plasma 25-hydroxyvitamin D (25OHD) deficiency, poor sleep quality, and night-time eating, have been independently associated with adverse pregnancy outcomes, but their inter-relationships are yet to be evaluated. We aimed to investigate the associations between maternal plasma 25OHD status and sleep quality and circadian eating patterns during pregnancy. Data on pregnant women (*n* = 890) from a prospective cohort (Growing Up in Singapore Towards healthy Outcomes) were analyzed. Plasma 25OHD concentration was measured, while the Pittsburgh sleep quality index (PSQI) and 24-h dietary recall were administered to women at 26–28 weeks’ gestation. Plasma 25OHD status was defined as sufficient (>75 nmol/L), insufficient (50–75 nmol/L), or deficient (<50 nmol/L). Poor sleep quality was defined by a total global PSQI score >5. Predominantly day-time (pDT) and predominantly night-time (pNT) were defined according to consumption of greater proportion of calories (i.e., >50%) from 07:00–18:59 and from 19:00–06:59, respectively. After adjustment for confounders, women with plasma 25OHD deficiency had higher odds of poor sleep quality (odds ratio (OR) 3.49; 95% confidence interval (CI) 1.84–6.63) and pNT eating (OR: 1.85; 95% CI 1.00–3.41) than those who were 25OHD sufficient. Our findings show the association of maternal plasma 25OHD deficiency with poor sleep quality and pNT eating at mid-pregnancy.

## 1. Introduction

Human behavioral rhythms involve rest and activity cycles such as sleep/wakefulness and fasting/feeding, which are synchronized with environmental signals, particularly dark/light intervals, across a 24-h time period [1]. The synchronization process involves hierarchical mechanisms where the central circadian clock at the hypothalamic suprachiasmatic nucleus (SCN) is entrained to sunlight via the retina, and subsequently influences peripheral oscillators through hormonal and neural pathways [2]. When daily central and peripheral rhythms are chronically disrupted or misaligned, the consequence of these circadian disruptions may be the development of metabolic disease [3]. Recent experimental studies [4,5,6] have suggested that the central circadian clock may also be regulated by another solar zeitgeber, vitamin D. Both 1,25-dihydroxycholecalciferol receptor (VDR) and 1α-hydroxylase (1α-OHase) were found to be widespread in human brain [4]. In adipose-derived stem cells, 1,25-dihydroxycholecalciferol was able to control the expression of circadian clock genes (i.e., BMAL1 and PER2) [5]. In addition, daily plasma 25-hydroxyvitamin D (25OHD) levels were reported to exhibit circadian oscillation [6]. Therefore, it is possible that vitamin D, otherwise known as the ‘sunshine vitamin’ [7], may influence circadian rhythms and related health outcomes.

Several studies have reported that 25OHD deficiency [8] and disruptions in behavioral circadian rhythms such as sleep disorders [9], and predominantly night-time eating [10], are prevalent among pregnant women. These trends are of concern because 25OHD deficiency [7,11], sleep disorders [12], and night-time eating [10,13], have been linked with pregnancy complications including impaired glucose tolerance, gestational diabetes mellitus, and preeclampsia, which can in turn impair both mothers’ and offspring’s long term health [14,15]. It has been widely reported that participants who were deficient in 25OHD were found to be at higher risk of sleep disorders [16,17,18,19,20], but these findings were limited to older adult populations (mean age: 42–68 years), and may not be generalizable to reproductive-aged populations. To our best knowledge, only one study was conducted among pregnant women, but the sample size was modest (*n* = 87), and their result showing poorer sleep quality in 25OHD deficient women versus non-deficient women did not reach statistical significance [21]. Moreover, no study has yet examined vitamin D in relation to other circadian behaviors such as eating patterns. Therefore, further investigation on vitamin D and behavioral circadian rhythms among pregnant women is warranted to facilitate the design of intervention strategies, to prevent pregnancy complications and to reduce future health burdens.

Geographical latitude has been shown to influence circulating 25OHD concentrations throughout the year [22,23] as well as several sleep traits [24], due to seasonal variations in day length. We analyzed data from a prospective cohort study conducted in Singapore, where the daylight length of approximately 12 h is relatively constant all year round. The aim of this study was to investigate the associations of 25OHD status with sleep quality and circadian eating patterns among pregnant Asian women. We hypothesized that mothers who were deficient in 25OHD were more likely to have poor sleep quality, and demonstrate a predominantly night-time food intake pattern during pregnancy.

## 2. Methods

### 2.1. Study Design and Participants

A total of 1152 women with singleton pregnancy, who conceived naturally, were recruited to an ongoing prospective cohort study, Growing Up in Singapore Towards healthy Outcomes (GUSTO), during the first trimester of pregnancy (≤14 weeks of gestation by ultrasound dating) at KK Women’s and Children’s Hospital and National University Hospital in Singapore between June 2009 and September 2010, as detailed in Soh et al. [25]. They were at least 18 years of age, citizens or permanent residents and had homogeneous parental ethnic groups (Chinese, Malay or Indian, who have increasingly darker skin tones from yellow tan, medium brown to dark brown). Pregnant women who were on chemotherapy or psychotropic drugs or who had type 1 diabetes mellitus were excluded from the beginning of the study. Informed written consent was obtained from all participants. This study was conducted according to the guidelines laid down in the Declaration of Helsinki. Ethical approval was obtained from the Centralised Institutional Review Board of SingHealth (reference 2009/280/D) and the Domain Specific Review Board of Singapore National Healthcare Group (reference D/09/021). This study was registered at www.clinicaltrials.gov as NCT01174875.

### 2.2. Maternal Characteristics

Pregnant women were interviewed at the recruitment visit and at 26–28 weeks’ gestation, to record their socio-demographic characteristics, past medical history (i.e., Type 2 diabetes, hypertension), and lifestyles during pregnancy (i.e., night shift status, vitamin D supplementation, physical activity). Vitamin D supplementation status was based on the reported intake of vitamin D only supplements or multivitamins containing vitamin D. Total score of physical activity was calculated from the summation of the duration (in minutes) and frequency (days) of three types activities (i.e., light-moderate, moderate and vigorous intensity), and expressed in metabolic equivalent task (MET-minutes/week) to classify women into: (i) not highly active (<3000 MET-minutes/week); and (ii) highly active (≥3000 MET-minutes/week) [26,27]. Additionally, pregnant women self-administered the Edinburgh Postnatal Depression Scale (EPDS) at 26–28 weeks’ gestation to assess their prenatal depression status [28,29]. Maternal height was measured using a stadiometer (SECA 213, Hamburg, Germany). Self-reported pre-pregnancy weight was collected at recruitment visit, while maternal weight in early pregnancy was measured at ≤14 weeks’ gestation. Body Mass Index (BMI) was calculated as weight in kilograms (kg) divided by height in meters squared (m^2^). Since maternal BMI in early pregnancy was strongly correlated with pre-pregnancy BMI (*r* = 0.96, *p* < 0.001) and had a lower percentage of missing data (*n* = 29, 3.3%), it was used for analyses in this study. Serial maternal weight measurements during pregnancy were extracted from medical records to estimate an individual linear trajectory of gestational weight gain per week between 15 and 35 weeks’ gestation, using linear mixed-effects model with the Best Linear Unbiased Predictor [30].

### 2.3. Plasma 25OHD Measurements

At 26–28 weeks’ gestation, an overnight fasting blood sample was drawn. Plasma 25OHD was analyzed as 25-hydroxyergocalciferol and 25-hydroxycholecalciferol by isotope-dilution liquid chromatography–tandem mass spectrometry [31]. The intra- and inter-assay coefficient of variation (CV) for 25OHD_2_ and 25OHD_3_ were ≤10.3%, and the detection limit was <4 nmol/L for both metabolites. In this cohort, plasma 25OHD concentrations were mainly 25-hydroxycholecalciferol concentrations since 25-hydroxyergocalciferol concentrations were mostly either below detection limits or zero. We classified maternal plasma 25OHD status as: (i) sufficient (>75 nmol/L); (ii) insufficient (50–75 nmol/L) and (iii) deficient (<50 nmol/L) [32]. For additional analyses, we classified maternal plasma 25OHD status into: (i) sufficient (>75 nmol/L); and (ii) inadequate (≤75 nmol/L) [32,33]. However, we did not classify plasma 25OHD status using the Institute of Medicine’s (IOM) 2011 definition [34] due to the modest sample size in those with plasma 25OHD <30 nmol/L (*n* = 16).

### 2.4. Sleep Quality

The Pittsburg sleep quality index (PSQI) [35] was self-administered by mothers at 26–28 weeks’ gestation to assess their sleep quality and disturbances in the past one month. It consists of 19 items which are rated on a four-point scale (0–3) and grouped into seven components (sleep quality, sleep latency, sleep duration, habitual sleep efficiency, sleep disturbance, use of sleeping medications, and daytime dysfunction). The item scores in each component were summed and converted to component scores ranging from 0 (better) to 3 (worse) based on guidelines [35]. Total PSQI score was calculated as the summation of seven component scores ranging from 0 to 21, where higher score indicates worse condition. A score of >5 on total global PSQI score is indicative of poor sleep quality [35].

### 2.5. Circadian Eating Patterns

A 24 h dietary recall was administered to mothers by trained clinical staff at 26–28 weeks’ gestation using the five-stage, multiple-pass interviewing technique [36]. Daily energy and macronutrient intakes were estimated using a nutrient analysis software (Dietplan 7, Forestfield Software) with a food composition database of locally available foods [37]) and modifications made on inaccuracies found. For mixed dishes not found in the local database, nutrient analyses of recipes were conducted with the use of the nutrient software. For other food items not found in the database, nutrient information was obtained from either food labels or the United States Department of Agriculture national nutrient database [38]. 

Sunlight is a strong environmental signal for human circadian clock [39]. In Singapore (1.3° North, 103.8° East) [40], sunrise (~07:00) and sunset (~19:00) times remain fairly constant throughout the year [41]. In this study, maternal eating patterns were classified as (i) predominantly day-time (pDT) eating, if women consumed more than 50% of total energy intake during daylight period from 07:00 to 18:59; and (ii) predominantly night-time (pNT) eating, if women consumed more than 50% of total energy intake during the nightfall hours from 19:00 to 06:59 [10].

### 2.6. Statistical Analyses

Differences in maternal characteristics between the excluded and included participants, and across 25OHD status, were compared using Fisher’s exact tests for categorical variables and One-way Analysis of Variance (ANOVA) for continuous variables. The associations of maternal plasma 25OHD status with sleep quality and circadian eating patterns were examined using logistic regression, with 25OHD status as the key independent variable. Potential confounders were selected based on their biological importance, or known confounding factors from review of the literature. While the direct impact of biological factors (ethnicity, age, BMI, gestational weight gain) on plasma 25OHD concentration is well established (23), it is possible that social (education, household income, parity) and behavioral factors (night shift status, physical activity, depression) may influence plasma 25OHD concentration [42,43]. Moreover, biological, social, and behavioral factors may affect mothers’ sleep comfort and preferences in meal scheduling. Therefore, multivariate analyses in this study were adjusted for ethnicity, age, BMI in early pregnancy, education, household income, parity, night shift status, physical activity, total EPDS score, and gestational weight gain per week. Additional analyses were computed to test the association between maternal plasma 25OHD status and dual disruptions in circadian behavior (i.e., both poor sleep quality and pNT eating which may alter the circadian time structure of human beings [44]). The associations of maternal plasma 25OHD concentrations with sleep quality, circadian eating patterns, and dual behavioral disruption, were also tested using logistic regression to explore dose-effect relationships. All statistical analyses were performed using Stata 13.1 (StataCorp, College Station, TX, USA). A two-tailed *p* value of <0.05 was considered to be statistically significant.

## 3. Results

### 3.1. Study Participants

This study analyzed more than three-quarters (*n* = 890, 77.3%) of 1152 pregnant women from the GUSTO cohort. The reasons for exclusion were missing data on (i) plasma 25OHD concentration (*n* = 210, 18.2%); and (ii) both sleep quality and circadian eating patterns (*n* = 52, 4.5%) (Figure S1). Most of the maternal characteristics such as ethnicity, age, BMI at ≤14 weeks’ gestation, physical activity, parity, night shift status, past medical history, vitamin D supplementation, gestational weight gain per week, and total EPDS score, were comparable (all *p* > 0.05) between the excluded and included participants. However, the included participants were more likely to be older (*p* = 0.002), attained higher education level (*p* < 0.001) and had greater household monthly income (*p* = 0.001) than those excluded (Table S1).

In the present study, over half of the maternal plasma 25OHD status were classified as sufficient (*n* = 527, 59.2%), followed by insufficient (*n* = 244, 27.4%) and deficient (*n* = 119, 13.4%). Table 1 shows the comparisons of maternal characteristics across plasma 25OHD status. Women classified as insufficient and deficient 25OHD were younger (*p* < 0.001), heavier at ≤14 weeks’ gestation (*p* = 0.002), as well as less likely to be Chinese (*p* < 0.001), receive tertiary education (*p* = 0.012) and consume vitamin D supplements (*p* < 0.001), compared to women with sufficient 25OHD. No differences were observed in other characteristics across plasma 25OHD status.

Table 2 shows the comparisons of maternal sleep, eating and combined sleep and eating traits across plasma 25OHD status during pregnancy. Pregnant women with 25OHD deficiency had mean bed times later than those with 25OHD sufficiency and insufficiency (*p* = 0.001). They also had the highest mean PSQI component scores in sleep latency (*p* = 0.010), sleep duration (*p* < 0.001), habitual sleep efficiency (*p* = 0.001), sleep disturbances (*p* = 0.004) and daytime dysfunction (*p* = 0.012), as well as highest mean total PSQI score (*p* < 0.001). There were no differences in scores for subjective sleep quality and use of sleeping medication among all three groups (*p* > 0.05). Compared to pregnant women with sufficient 25OHD, those deficient in 25OHD were more likely to have poor sleep quality (*p* < 0.001) and pNT eating (*p* = 0.049). These women were also more likely to have dual disruptions in circadian behavior (*p* < 0.001). 

### 3.2. Plasma 25OHD Status and Behavioral Circadian Rhythms

Table 3 shows the associations of plasma 25OHD status with sleep quality and circadian eating patterns. Compared to pregnant women with sufficient 25OHD, those deficient in 25OHD had more than three times higher odds of having poor sleep quality (Odds ratio (OR): 3.19; 95% Confidence Interval (CI): 1.89, 5.39), and almost two times higher odds of pNT eating (OR: 1.85; 95% CI: 1.10, 3.13). They also had six times higher odds of having both poor sleep quality and pNT eating (OR: 6.01; 95% CI: 2.55, 14.16). On the other hand, pregnant women with insufficient 25OHD were associated with poor sleep quality (OR: 1.47; 95% CI: 1.00, 2.15), but not with pNT eating nor dual circadian behavioral disruptions. When adjusted for confounders, similar findings were found for 25OHD deficiency, although the association of 25OHD deficiency with pNT eating was near to statistical significance. No associations of 25OHD insufficiency with poor sleep quality, pNT eating and dual circadian behavioral disruptions were obtained (Table 3). Further analyses showed that pregnant women with 25OHD inadequacy were associated with higher odds of having poor sleep quality (adjusted OR: 2.02; 95% CI: 1.28, 3.20; *p* = 0.003) and dual disruptions in circadian behavioral rhythms (adjusted OR: 2.74; 95% CI: 1.02, 7.34; *p* = 0.045) but not with pNT eating (adjusted OR: 1.19; 95% CI: 0.74, 1.93; *p* = 0.466). Similarly, higher plasma 25OHD concentrations (per 10 nmol/L increment) were associated with lower odds of poor sleep quality (adjusted OR: 0.90; 95% CI: 0.83, 0.98; *p* = 0.019) and dual circadian behavioral disruptions (adjusted OR: 0.81; 95% CI: 0.66, 0.99; *p* = 0.040), but not with pNT eating (adjusted OR: 0.98; 95% CI: 0.90, 1.07; *p* = 0.682) (data not shown in table).

## 4. Discussion

This study provided new evidence that maternal plasma 25OHD status is associated with sleep quality and circadian eating patterns at mid-pregnancy in an Asian cohort from an equatorial country. Pregnant women who were plasma 25OHD deficient had higher odds of having poor sleep quality and pNT feeding, and even higher odds of having both poor sleep quality and pNT feeding, compared to those who were 25OHD sufficient. These findings remained consistent even after adjustment for biological, social, and behavioral factors, suggesting that plasma 25OHD concentration may be a biomarker of circadian behavioral status among pregnant women. 

Our findings are consistent with previous studies that associated serum 25OHD concentration with sleep traits in older adult populations living at more northerly latitudes. Among elderly participants (mean age: 68 years old), those who slept less than four hours had the lowest 25OHD levels in a Korean study [17], whereas those with 25OHD deficiency (<20 ng/mL) had the shortest sleep duration, lowest sleep efficiency and highest sleepiness scores in an American study [16]. A positive linear correlation of 25OHD level with sleep duration, but not total PSQI score, was also seen among pre- and post-menopausal women from Southern England [19]. Similarly, in middle-aged groups ranging from 42 to 48 years old in America, low 25OHD (<30 ng/mL) participants reported longer sleep latency [18], while negative and positive correlations between 25OHD level and excessive daytime sleepiness were observed among non-25OHD deficient (≥20 ng/mL), and 25OHD deficient (<20 ng/mL) participants, respectively [20]. 

Only one related study has been conducted among pregnant women (mean age: 30.4 years old) in Turkey, which showed that women at 36 weeks’ gestation in the serum 25OHD deficient group (<50 nmol/L) (*n* = 46) had poorer sleep quality and shorter sleep duration than the 25OHD non-deficient group (*n* = 41), but the associations were not statistically significant [21]. This Turkish study may also be limited by seasonal variation throughout the study period from Jan to July 2013 [21], due to its geographical location at a high latitude (39.9° North, 32.8° East) [40], which could lead to alternations in ultraviolet light exposure, and a larger peak-trough difference in serum 25OHD concentrations [22]. In contrast, our findings among a larger group of pregnant women were unlikely to be affected by seasonal changes, since the duration of day length, and sunrise and sunset times, are relatively constant all year round [41] in Singapore [40].

Since sleep and eating are key human behaviors that determine long term health [45], and eating disorders may be sleep-related [46], we further examined the 24-h feeding patterns of pregnant women, beyond traditionally studied sleep characteristics and practices [16,17,18,19,20,21]. We classified daily eating patterns into predominantly day-time and predominantly night-time eating in this study, on the basis that a significantly increased intake in the evening/night-time is a core criterion of circadian disorder known as night eating syndrome [47]. We demonstrated that pregnant women who were deficient in plasma 25OHD were associated with larger energy consumption during night-time hours (19:00–06:59), although the association was of borderline statistical significance. Notably, these women were associated with higher odds for dual circadian behavioral disruption, suggesting that both sleep quality and feeding patterns should be considered when assessing circadian behavior, although this finding needs to be confirmed with a larger sample size. The examination of combined sleep and eating patterns was targeted at dual rather than single disruptions, as we also aimed to study the additive effect of circadian behavioral disruptions. 

The underlying mechanism to explain our findings is currently unknown. However, we hypothesize that plasma 25OHD deficiency may contribute to circadian behavioral disruptions in pregnant women. One possible explanation is that the low level of 25OHD present in blood is unable to fully activate the central circadian clock that regulates peripheral circadian clocks, ultimately leading to desychronization of peripheral rhythms [2] with natural day/night cycles. The possible function of vitamin D at the central clock is supported by recent discoveries [4,5]. Besides being present in the kidneys, the enzyme 1α-OHase is found in the human brain and converts 25OHD in the bloodstream to 1,25-dihydroxyvitamin D (1,25OHD), that then activates the VDR [4], which is a ligand transcription factor that regulates gene expression [48], probably including circadian clock genes. Alternatively, 1,25OHD may also directly interact with circadian clock genes to control their gene expression [5]. Given that sunlight drives both vitamin D synthesis [7] and circadian rhythms [2], it is possible that vitamin D transduces light signals to regulate circadian rhythms [49]. Another explanation for the present findings may be that plasma 25OHD concentrations may be a surrogate measure of retinal sunlight exposure [50] which entrains the human circadian clock [39].

Since data in this study were collected in a cross-sectional manner, reverse causality is possible. Pregnant women with unhealthy lifestyles (poor sleep quality, predominantly night-time eating) may tend to stay indoors, have less sunlight exposure and consequently lower endogenous vitamin D production. Alternatively, poor sleep quality and predominantly night-time eating may cause 25OHD deficiency by disrupting hormonal rhythms and metabolism [51], thereby impairing gastrointestinal absorption of vitamin D and/or functions of kidney and liver that are involved in 25OHD synthesis [23]. These potential causal pathways may partially explain the negative findings in previous studies aiming to determine the benefits of vitamin D supplementation [50]. Participants who received vitamin D supplements and did not show improved disease prevention and treatment [50], may have failed to increase their plasma 25OHD concentration as a result of disrupted behavioral circadian rhythms. Similarly, the high prevalence of low vitamin D levels despite abundant sunlight exposure among those living at lower latitudes [23] may be mediated by circadian behavioral disruptions. Therefore, we postulate that factors influencing plasma 25OHD level may not be limited to variation in exposure to ultraviolet radiation, skin pigmentation, age, obesity, and chronic diseases such as kidney disease, liver disease, and granulomatous disorders [23], but may also include behavioral circadian rhythms. 

The present study has several methodological strengths which increase the reliability of our findings. We included a large sample of otherwise healthy pregnant women who live in a localized equatorial environment, employed a reliable and accurate method to measure 25OHD concentrations [52], and accounted for a broad range of confounders, including variation in skin tone as represented by ethnicity in the analyses. Nevertheless, our findings should be interpreted cautiously. We did not assess plasma 25OHD levels throughout the day and during the entire course of pregnancy in our cohort of pregnant women. We noted several differences in characteristics between the excluded and included in this study, which have been adjusted for in multivariate analyses. Data from the PSQI and 24-h dietary recall were based on self-reported questionnaires but the PSQI has been validated and the 24-h dietary recall was administered using a standardized protocol to minimize potential measurement errors. Although the 24-h dietary patterns may not be representative of usual food intake, this is less likely to affect our findings since poor measurement from single use of 24-h recall tends to bias findings towards the null. Additionally, the cutoff points for plasma 25OHD concentrations, poor sleep quality among pregnant women, and the classification of circadian feeding patterns are yet to be validated. Finally, the actual duration of sunlight and artificial light exposure were not recorded, and thus cannot be accounted for in the analyses. 

## 5. Conclusions

In conclusion, maternal plasma 25OHD deficiency was associated with poor sleep quality and pNT feeding during the late-second trimester of pregnancy in Singapore. These findings generate possibilities for future research. To understand the causal direction of our findings, similar studies should be replicated using a longitudinal design and in randomized trials. Gene expression studies of circadian clock genes across plasma 25OHD status can be performed. Further studies should evaluate whether increasing plasma 25OHD levels in deficient individuals can induce improvements in human circadian behavior, or if improving circadian behavior can itself increase plasma 25OHD levels.

## Figures and Tables

**Table 1:** Comparisons of maternal characteristics across plasma 25OHD status ^1^.

Maternal Characteristics	Plasma 25OHD Status			
	Sufficient (>75 nmol/L) (*n* = 527, 59.2%)	Insufficient (50–75 nmol/L) (*n* = 244, 27.4%)	Deficient (<50 nmol/L) (*n* = 119, 13.4%)	*p* Value ^2^
Ethnicity				<0.001
Chinese	367 (74.9)	96 (19.6)	27 (5.5)	
Malay	90 (38.1)	90 (38.1)	56 (23.7)	
Indian	70 (42.7)	58 (35.4)	36 (22.0)	
Education				0.012
None/primary/secondary	159 (60.2)	62 (23.5)	43 (16.3)	
Post-secondary	163 (53.1)	103 (33.6)	41 (13.4)	
Tertiary	199 (64.4)	77 (24.9)	33 (10.7)	
Household monthly income				0.054
<SGD2000	71 (59.2)	30 (25.0)	19 (15.8)	
SGD2000-5999	262 (55.5)	141 (29.9)	69 (14.6)	
≥SGD6000	158 (65.3)	63 (26.0)	21 (8.7)	
Physical activity				0.896
Not highly active (<3000 MET-minutes/week)	422 (59.4)	192 (27.0)	96 (13.5)	
Highly active (≥3000 MET-minutes/week)	97 (57.7)	48 (28.6)	23 (13.7)	
Parity				0.717
Nulliparous	223 (59.6)	105 (28.1)	46 (12.3)	
Multiparous	304 (58.9)	139 (26.9)	73 (14.1)	
Night shift status				0.275
No	500 (59.2)	235 (27.8)	110 (13.0)	
Yes	27 (60.0)	9 (20.0)	9 (20.0)	
Past medical history ^3^				0.115
No	507 (59.4)	230 (26.9)	117 (13.7)	
Yes	11 (57.9)	8 (42.1)	0 (0.0)	
Vitamin D supplementation				<0.001
No	67 (46.5)	42 (29.2)	35 (24.3)	
Yes	424 (64.2)	173 (26.2)	63 (9.5)	
Plasma 25OHD concentration (nmol/L)	99.1 ± 18.5	63.5 ± 7.4	39.0 ± 7.5	<0.001
Gestational weight gain per week	0.47 ± 0.11	0.46 ± 0.11	0.47 ± 0.11	0.815
Age (years)	31.2 ± 5.0	29.9 ± 4.9	29.1 ± 5.3	<0.001
BMI at ≤14 weeks’ gestation (kg/m^2^)	23.2 ± 4.3	24.1 ± 5.0	24.6 ± 5.2	0.002
Total EPDS score	7.2 ± 4.4	7.5 ± 4.5	8.3 ± 4.5	0.066

^1^ Total sample size ranged from 804 to 890 due to missing values; ^2^ based on Fisher’s exact test and One-way Analysis of Variance (ANOVA) as appropriate; ^3^ defined as type 2 diabetes or hypertension; 25OHD: 25-hydroxyvitamin D; BMI: body mass index; EPDS: Edinburgh Postnatal Depression Scale; MET: metabolic equivalent task; SGD: Singapore dollars.

**Table 2:** Comparisons of prenatal sleep, eating and combined sleep and eating traits across plasma 25OHD status.

Circadian Behavior	Plasma 25OHD Status			
	Sufficient (>75 nmol/L)	Insufficient (50–75 nmol/L)	Deficient (<50 nmol/L)	*p* Value ^1^
Sleep traits	*n* = 330	*n* = 162	*n* = 76	
Bed time (hour:minute) ^2^	23:09 ± 1:08	23:18 ± 1:38	23:47 ± 1:41	0.001
Wake time (hour:minute) ^2^	7:22 ± 1:45	7:35 ± 2:11	7:46 ± 2:22	0.182
Component 1—Subjective sleep quality	1.04 ± 0.60	1.08 ± 0.68	1.21 ± 0.73	0.115
Component 2—Sleep latency	1.05 ± 0.84	1.21 ± 0.96	1.36 ± 0.97	0.010
Component 3—Sleep duration	0.38 ± 0.72	0.50 ± 0.82	0.77 ± 1.05	<0.001
Component 4—Habitual sleep efficiency	0.45 ± 0.83	0.68 ± 1.02	0.84 ± 1.12	0.001
Component 5—Sleep disturbances	1.46 ± 0.57	1.55 ± 0.59	1.70 ± 0.68	0.004
Component 6—Use of sleeping medication	0.03 ± 0.23	0.08 ± 0.37	0.07 ± 0.31	0.199
Component 7—Daytime dysfunction	0.73 ± 0.59	0.74 ± 0.65	0.95 ± 0.74	0.012
Total PSQI score	5.10 ± 2.63	5.72 ± 3.03	7.04 ± 3.47	<0.001
Sleep quality				<0.001
Good	206 (64.8)	86 (27.0)	26 (8.2)	
Poor	124 (49.6)	76 (30.4)	50 (20.0)	
Eating trait	*n* = 488	*n* = 223	*n* = 104	
Circadian eating patterns				0.049
Predominantly day-time eating	420 (60.5)	194 (28.0)	80 (11.5)	
Predominantly night-time eating	68 (56.2)	29 (24.0)	24 (19.8)	
Combined sleep and eating traits	*n* = 177	*n* = 75	*n* = 31	
Dual disruptions in behavioral circadian rhythms				<0.001
Good sleep quality and predominantly day-time eating	158 (65.3)	66 (27.3)	18 (7.4)	
Poor sleep quality and predominantly night-time eating	19 (46.3)	9 (22.0)	13 (31.7)	

^1^ based on Fisher’s exact test and One-way Analysis of Variance (ANOVA) as appropriate; ^2^ The time when participants went to bed and woke up on a 24-h clock time; 25OHD: 25-hydroxyvitamin D; PSQI: Pittsburg sleep quality index.

**Table 3:** Associations of plasma 25OHD status with sleep quality, circadian eating patterns, and dual disruptions in behavioral circadian rhythms, during mid-pregnancy.

Circadian Behavior	Crude		Adjusted ^1^	
	OR (95% CI)	*p* Value	OR (95% CI)	*p* Value
Poor sleep quality				
25OHD status				
Sufficient (>75 nmol/L)	Reference		Reference	
Insufficient (50–75 nmol/L)	1.47 (1.00, 2.15)	0.048	1.51 (0.91, 2.50)	0.114
Deficient (<50 nmol/L)	3.19 (1.89, 5.39)	<0.001	4.14 (2.01, 8.51)	<0.001
Predominantly night-time eating				
25OHD status				
Sufficient (>75 nmol/L)	Reference		Reference	
Insufficient (50–75 nmol/L)	0.92 (0.58, 1.47)	0.738	0.96 (0.55, 1.65)	0.901
Deficient (<50 nmol/L)	1.85 (1.10, 3.13)	0.021	1.89 (0.99, 3.64)	0.055
Poor sleep quality and predominantly night-time eating				
25OHD status				
Sufficient (>75 nmol/L)	Reference		Reference	
Insufficient (50–75 nmol/L)	1.13 (0.49, 2.64)	0.770	1.33 (0.40, 4.39)	0.638
Deficient (<50 nmol/L)	6.01 (2.55, 14.16)	<0.001	9.74 (2.57, 36.90)	0.001

^1^ adjusted for ethnicity, age, education level, household income, BMI at ≤14 weeks’ gestation, night shift status, total EPDS score, parity, physical activity, gestational weight gain per week; 25OHD: 25-hydroxyvitamin D; BMI: body mass index; EPDS: Edinburgh Postnatal Depression Scale; CI: Confidence interval; OR: Odds ratio.

## Supplementary Materials

The following are available online at http://www.mdpi.com/2072-6643/9/4/340/s1, Figure S1: Flow chart of study inclusion, Table S1: Comparison of characteristics between excluded and included pregnant mothers.

## References

[1] Vitaterna M.H., Takahashi J.S., Turek F.W. (2001). Overview of circadian rhythms. Alcohol Res. Health.

[2] Dibner C., Schibler U., Albrecht U. (2010). The mammalian circadian timing system: Organization and coordination of central and peripheral clocks. Ann. Rev. Physiol..

[3] Voigt R.M., Forsyth C.B., Keshavarzian A. (2013). Circadian disruption: Potential implications in inflammatory and metabolic diseases associated with alcohol. Alcohol Res. Curr. Rev..

[4] Eyles D.W., Smith S., Kinobe R., Hewison M., McGrath J.J. (2005). Distribution of the vitamin D receptor and 1α-hydroxylase in human brain. J. Chem. Neuroanat..

[5] Gutierrez-Monreal M.A., Duran R.C.-D., Moreno-Cuevas J.E., Scott S.-P. (2014). A role for 1α; 25-dihydroxyvitamin D3 in the expression of circadian genes. J. Biol. Rhythms.

[6] Masood T., Kushwaha R.S., Singh R., Sailwal S., Pandey H., Varma A., Singh R.K., Cornelissen G. (2015). Circadian rhythm of serum 25 (OH) vitamin D, calcium and phosphorus levels in the treatment and management of type-2 diabetic patients. Drug Discov. Ther..

[7] Holick M.F. (2007). Vitamin D deficiency. N. Engl. J. Med..

[8] Johnson D.D., Wagner C.L., Hulsey T.C., McNeil R.B., Ebeling M., Hollis B.W. (2011). Vitamin D deficiency and insufficiency is common during pregnancy. Am. J. Perinatol..

[9] Mindell J.A., Cook R.A., Nikolovski J. (2015). Sleep patterns and sleep disturbances across pregnancy. Sleep Med..

[10] Loy S.L., Cheng T.S., Colega M.T., Cheung Y.B., Godfrey K.M., Gluckman P.D., Kwek K., Saw S.M., Chong Y-S., Padmapriya N. (2016). Predominantly night-time feeding and maternal glycaemic levels during pregnancy. Br. J. Nutr..

[11] Lapillonne A. (2010). Vitamin D deficiency during pregnancy may impair maternal and fetal outcomes. Med. Hypotheses.

[12] Facco F.L., Grobman W.A., Kramer J., Ho K.H., Zee P.C. (2010). Self-reported short sleep duration and frequent snoring in pregnancy: Impact on glucose metabolism. Am. J. Obstet. Gynecol..

[13] Chandler-Laney P.C., Schneider C.R., Gower B.A., Granger W.M., Mancuso M.S., Biggio J.R. (2016). Association of late-night carbohydrate intake with glucose tolerance among pregnant African American women. Matern. Child. Nutr..

[14] Godfrey K.M., Barker D.J. (2001). Fetal programming and adult health. Public Health Nutr..

[15] Sattar N., Greer I.A. (2002). Pregnancy complications and maternal cardiovascular risk: Opportunities for intervention and screening?. BMJ.

[16] Bertisch S., Sillau S., de Boer I., Szklo M., Redline S. (2015). 25-Hydroxyvitamin D Concentration and Sleep Duration and Continuity: Multi-Ethnic Study of Atherosclerosis. Sleep.

[17] Kim J.H., Chang J.H., Kim D.Y., Kang J.W. (2014). Association between self-reported sleep duration and serum vitamin D level in elderly Korean adults. J. Am. Geriatr. Soc..

[18] Shiue I. (2013). Low vitamin D levels in adults with longer time to fall asleep: US NHANES; 2005–2006. Int. J. Cardiol..

[19] Darling A., Skene D., Lanham-New S. (2011). Preliminary evidence of an association between vitamin D status and self-assessed sleep duration but not overall sleep quality: Results from the D-FINES study of South Asian and Caucasian pre-and post-menopausal women living in Southern England. Proc. Nutr. Soc..

[20] McCarty D.E., Reddy A., Keigley Q., Kim P.Y., Marino A.A. (2012). Vitamin D, race, and excessive daytime sleepiness. J. Clin. Sleep Med..

[21] Gunduz S., Kosger H., Aldemir S., Akcal B., Tevrizci H., Hizli D., Celik H.T. (2016). Sleep deprivation in the last trimester of pregnancy and inadequate vitamin D: Is there a relationship?. J. Chin. Med. Assoc..

[22] Shoben A.B., Kestenbaum B., Levin G., Hoofnagle A.N., Psaty B.M., Siscovick D.S., de Boer I.H. (2011). Seasonal variation in 25-hydroxyvitamin D concentrations in the cardiovascular health study. Am. J. Epidemiol..

[23] Tsiaras W.G., Weinstock M.A. (2011). Factors influencing vitamin D status. Acta Derm. Venereol..

[24] Friborg O., Bjorvatn B., Amponsah B., Pallesen S. (2012). Associations between seasonal variations in day length (photoperiod), sleep timing, sleep quality and mood: A comparison between Ghana (5) and Norway (69). J. Sleep Res..

[25] Soh S.-E., Tint M.T., Gluckman P.D., Godfrey K.M., Rifkin-Graboi A., Chan Y.H., Stünkel W., Holbrook J.D., Kwek K., Chong Y.-S. (2014). Cohort Profile: Growing Up in Singapore Towards healthy Outcomes (GUSTO) birth cohort study. Int. J. Epidemiol..

[26] Padmapriya N., Shen L., Soh S.-E., Shen Z., Kwek K., Godfrey K.M., Gluckman P.D., Chong Y.-S., Saw S.-M., Müller-Riemenschneider F. (2015). Physical activity and sedentary behavior patterns before and during pregnancy in a multi-ethnic sample of Asian women in Singapore. Matern. Child. Health J..

[27] International Physical Acitivty Questionnaire (IPAQ) Guidelines for Data Processing and Analysis of the International Physical Acitivty Questionniare (IPAQ)—Short and Long Forms. https://sites.google.com/site/theipaq/scoring-protocol.

[28] Gibson J., McKenzie-McHarg K., Shakespeare J., Price J., Gray R. (2009). A systematic review of studies validating the Edinburgh Postnatal Depression Scale in antepartum and postpartum women. Acta Psychiatr. Scand..

[29] Cox J.L., Holden J.M., Sagovsky R. (1987). Detection of postnatal depression. Development of the 10-item Edinburgh Postnatal Depression Scale. Br. J. Psychiatry.

[30] Cheung Y.B. (2014). Statistical Analysis of Human Growth and Development.

[31] Maunsell Z., Wright D.J., Rainbow S.J. (2005). Routine isotope-dilution liquid chromatography-tandem mass spectrometry assay for simultaneous measurement of the 25-hydroxy metabolites of vitamins D2 and D3. Clin. Chem..

[32] Wei S.Q., Qi H.P., Luo Z.C., Fraser W.D. (2013). Maternal vitamin D status and adverse pregnancy outcomes: A systematic review and meta-analysis. J. Matern. Fetal Neonatal Med..

[33] Aghajafari F., Nagulesapillai T., Ronksley P., Tough S., O’Beirne M., Rabi D. (2013). Association between maternal serum 25-hydroxyvitamin D level and pregnancy and neonatal outcomes: Systematic review and meta-analysis of observational studies. BMJ.

[34] Institute of Medicine, Food and Nutrition Board (2010). Dietary Reference Intakes for Calcium and Vitamin D.

[35] Buysse D.J., Reynolds C.F., Monk T.H., Berman S.R., Kupfer D.J. (1989). The Pittsburgh Sleep Quality Index: A new instrument for psychiatric practice and research. Psychiatry Res..

[36] Conway J.M., Ingwersen L.A., Vinyard B.T., Moshfegh A.J. (2003). Effectiveness of the US Department of Agriculture 5-step multiple-pass method in assessing food intake in obese and nonobese women. Am. J. Clin. Nutr..

[37] Singapore Health Promotion Board Singapore: Healthy Living and Disease Prevention Information. http://www.hpb.gov.sg/HOPPortal/.

[38] United States Department of Agriculture (USDA) NDL/FNIC Food Composition Database. USDA National Nutrient Database for Standard Reference. http://ndb.nal.usda.gov/.

[39] Wright K.P., McHill A.W., Birks B.R., Griffin B.R., Rusterholz T., Chinoy E.D. (2013). Entrainment of the human circadian clock to the natural light-dark cycle. Curr. Biol..

[40] National Geospatial-Intelligence Agency Geonames WMS Viewer. http://geonames.nga.mil/namesviewer/.

[41] Astronomical Applications Department Sun or Moon Rise/Set Table for One Year.

[42] Joh H.K., Lim C.S., Cho B. (2015). Lifestyle and dietary factors associated with serum 25-hydroxyvitamin D levels in Korean young adults. J. Korean Med. Sci..

[43] Forrest K.Y.Z., Stuhldreher W.L. (2011). Prevalence and correlates of vitamin D deficiency in US adults. Nutr. Res..

[44] Smolensky M.H., Hermida R.C., Reinberg A., Sackett-Lundeen L., Portaluppi F. (2016). Circadian disruption: New clinical perspective of disease pathology and basis for chronotherapeutic intervention. Chronobiol. Int..

[45] Yang Z.Y., Yang Z., Zhu L., Qiu C. (2011). Human behaviors determine health: Strategic thoughts on the prevention of chronic non-communicable diseases in China. Int. J. Behav. Med..

[46] Inoue Y. (2015). Sleep-related eating disorder and its associated conditions. Psychiatry Clin. Neurosci..

[47] Vander Wal J.S. (2012). Night eating syndrome: A critical review of the literature. Clin. Psychol. Rev..

[48] Pike J.W., Meyer M.B. (2012). The vitamin D receptor: New paradigms for the regulation of gene expression by 1,25-dihydroxyvitamin D3. Rheum. Dis. Clin. N. Am..

[49] Lucock M., Jones P., Martin C., Beckett E., Yates Z., Furst J., Veysey M. (2015). Vitamin D: Beyond metabolism. J. Evid. Based Complement. Altern. Med..

[50] Hart P.H. (2012). Vitamin D supplementation, moderate sun exposure, and control of immune diseases. Discov. Med..

[51] Kim T.W., Jeong J.H., Hong S.C. (2015). The impact of sleep and circadian disturbance on hormones and metabolism. Int. J. Endrocrinol..

[52] Hollis B.W. (2008). Measuring 25-hydroxyvitamin D in a clinical environment: Challenges and needs. Am. J. Clin. Nutr..

