# Supplementary material for: Plasma Vitamin D Deficiency Is Associated with Poor Sleep Quality and Night-Time Eating at Mid-Pregnancy in Singapore
Source: Nutrients. 2017 Mar 29;9(4):340. doi: 10.3390/nu9040340 (PMC5409679; doi:10.3390/nu9040340)
Supplement: Supplementary file 1 [file nutrients-09-00340-s001.docx]

Supplementary Figure S1: Flow chart of study inclusion

Dual disruptions in behavioral circadian rhythms (n=283)

Single adverse circadian behaviors (n=210)

Missing data either on eating or sleep behavior (n=397)

Missing data on both eating and sleep behavior (n=52)

Missing data on plasma 25-hydroxycitamin D (n=210)

Data on sleep quality (n=568)

890

Data on circadian eating pattern (n=815)

942

Recruited into GUSTO cohort (n=1152)

| Supplementary Table S1: Comparison of characteristics between excluded and included pregnant mothers | | | |
| --- | --- | --- | --- |
|  | Excluded  (n=262) | Included  (n=890) | P value^a^ |
| Ethnicity (n, %) |  |  | 0.502 |
| Chinese | 136 (51.9) | 490 (55.1) |  |
| Malay | 79 (30.2) | 236 (26.5) |  |
| Indian | 47 (17.9) | 164 (18.4) |  |
| Education (n, %) |  |  | <0.001 |
| None/ primary/ secondary | 104 (40.5) | 264 (30.0) |  |
| Post-secondary | 95 (37.0) | 307 (34.9) |  |
| Tertiary | 58 (22.6) | 309 (35.1) |  |
| Household monthly income (n, %) |  |  | 0.001 |
| <SGD2000 | 57 (23.6) | 120 (14.4) |  |
| SGD2000-5999 | 137 (56.6) | 472 (56.6) |  |
| ≥SGD6000 | 48 (19.8) | 242 (29.0) |  |
| Physical activity (n, %) |  |  | 0.614 |
| Not highly active | 164 (82.8) | 710 (80.9) |  |
| Highly active | 34 (17.2) | 168 (19.1) |  |
| Parity (n, %) |  |  | 0.234 |
| Nulliparous | 92 (46.7) | 374 (42.0) |  |
| Multiparous | 105 (53.3) | 516 (58.0) |  |
| Night shift status (n, %) | 5 (2.5) | 45 (5.1) | 0.136 |
| Past medical history (n, %) | 6 (2.3) | 19 (2.2) | 0.812 |
| Vitamin D supplementation (n, %) | 146 (84.4) | 660 (82.1) | 0.510 |
| Gestational weight gain per week | 0.47 ± 0.11 | 0.47 ± 0.11 | 0.769 |
| Age (years) | 29.4 ±5.5 | 30.6 ± 5.1 | 0.002 |
| BMI at ≤14 weeks’ gestation (kg/m^2^) | 24.0 ± 5.4 | 23.6 ± 4.7 | 0.383 |
| Total EPDS score | 7.5 ± 4.7 | 7.4 ± 4.5 | 0.775 |
| BMI=body mass index, EPDS=Edinburgh Postnatal Depression Scale  ^a^P value was based on Fisher's exact test and One-way Analysis of Variance (ANOVA) as appropriate | | | |
